# Classic Hodgkin Lymphoma: Biology-Driven Disease Model

**DOI:** 10.3390/cancers18213592

**Published:** 2026-11-09

**Authors:** Antonino Carbone, Annunziata Gloghini

**Affiliations:** 1 Centro di Riferimento Oncologico, Istituto di Ricovero e Cura a Carattere Scientifico, National Cancer Institute, 33081 Aviano, Italy; 2 Former Senior Scientist at the Department of Advanced Diagnostics, Fondazione IRCCS, Istituto Tumori Milano, 20133 Milano, Italy; annunziata.gloghini@istitutotumori.mi.it

**Keywords:** classic Hodgkin lymphoma, tumor microenvironment, molecular subtypes, immune evasion, circulating tumor DNA, spatial biology, systemic disease, precision immunopathology

## Abstract

Background/Objectives: Classic Hodgkin lymphoma (cHL) is a unique lymphoid neoplasm in which rare malignant Hodgkin and Reed–Sternberg (HRS) cells orchestrate an extensive, spatially organized immune and inflammatory microenvironment that determines diagnosis, prognosis, and therapeutic sensitivity. This review summarizes recent advances in the biopathology, diagnosis, and prognostic stratification of classic Hodgkin lymphoma, with particular emphasis on the emerging molecular subtyping framework proposed by Aoki and colleagues, which is not yet clinically validated, and on the proposed concept of classic Hodgkin lymphoma as a systemic immunobiological disease. Methods: In this narrative review, we examined the literature on classic Hodgkin lymphoma biology, the tumor immune microenvironment, diagnostic and prognostic biomarkers, and molecular classification, prioritizing studies published through to mid-2026 and distinguishing established evidence from observational associations, preclinical findings, meeting abstracts, ongoing trials, and hypotheses, including single-cell, spatial, and liquid biopsy technologies, and integrating the authors’ own recent work on immune and inflammatory biomarkers and on the systemic immunobiological features of classic Hodgkin lymphoma. Results: Hodgkin and Reed–Sternberg cells sustain disease through constitutive NF-κB and JAK/STAT signaling, 9p24.1-driven PD-L1/PD-L2 overexpression, and secretion of chemokines and cytokines that recruit and functionally reprogram CD4+ T cells, regulatory T cells, macrophages, and stromal cells. Multidimensional profiling integrating malignant-cell genomics, spatial transcriptomics, and imaging mass cytometry has led to the proposal of four molecular subtypes of classic Hodgkin lymphoma (CST, CN913, STB, and CN2P), each defined by distinct genomic drivers and immune architecture, a hypothesis-generating framework that awaits prospective validation. Classic Hodgkin lymphoma has been proposed to be a systemic immunobiological disorder, with circulating tumor DNA, thymus and activation-regulated chemokine (TARC/CCL17), and peripheral immune signatures associated with disease activity beyond the lymph node; these markers are promising but remain investigational. Diagnosis is evolving from morphology-based assessment toward precision immunopathology integrating spatial and digital pathology, while prognosis is progressively incorporating tissue-based, circulating, spatial, and molecular biomarkers into integrated risk models that are, for the most part, still investigational and are being tested in biomarker-directed clinical trials. Conclusions: The convergence of molecular subtyping, spatial immunobiology, systemic disease biology, and liquid biopsy may reshape the diagnostic and prognostic landscape of classic Hodgkin lymphoma, suggesting a potential transition from anatomically defined, morphology-based assessment toward integrated, biology-driven precision medicine. This transition has unfolded stepwise, from tissue-based immunophenotyping and spatial biomarkers, through circulating markers such as ctDNA and TARC/CCL17, to genomically defined molecular subtypes now being linked to systemic immune readouts and tested in biomarker-directed trials. Prospective validation, assay standardization, and evidence of clinical utility are required before these approaches can enter routine practice.

## Simple Summary

Classic Hodgkin lymphoma is a cancer of the lymph nodes in which rare malignant cells are surrounded by large numbers of immune cells that they recruit and reprogram to support their own survival. This review describes how new technologies that analyze tumor genes, the spatial organization of cells within tissue, and tumor-derived material circulating in the blood are changing our understanding of the disease. A recent study has proposed four molecular subtypes with distinct biological features; this classification is promising but remains experimental and has not yet been validated in prospective clinical studies. Evidence is also accumulating that the disease affects the immune system beyond the lymph node, and we discuss this as a proposed model that complements, rather than replaces, current staging. Blood-based tests, such as circulating tumor DNA and the chemokine TARC, are being investigated for disease monitoring but are not yet standard tools for treatment decisions. Throughout, we distinguish established knowledge from clinical observations, laboratory findings, and hypotheses, to help readers judge how these advances may eventually support more precise diagnosis, risk assessment, and personalized treatment.

## 1. Introduction

Classic Hodgkin lymphoma (cHL) is a paradigmatic example of a malignancy whose clinical behavior is dictated less by the intrinsic genetics of its neoplastic clone than by the cellular and molecular ecosystem that surrounds it [1,2]. Malignant Hodgkin and Reed–Sternberg (HRS) cells typically account for only 1–10% of the total cellularity of an involved lymph node, the remainder being composed of a florid, non-random infiltrate of T and B lymphocytes, macrophages, eosinophils, mast cells, and stromal cells that HRS cells actively recruit, spatially organize, and functionally reprogram [1,3]. This extraordinary imbalance between tumor and microenvironment underlies both the diagnostic peculiarity of cHL and its exceptional responsiveness to immune checkpoint blockade, and it has made cHL one of the most informative models for studying tumor–immune interactions in oncology [2,3].

Over the past two years, three converging lines of evidence have substantially updated this picture. First, our own recent work has provided an integrated overview of how immune and inflammatory biomarkers are reshaping the diagnostic and prognostic assessment of cHL [4], and has proposed, as a conceptual model, a reinterpretation of cHL as a systemic immunobiological disorder in which HRS-derived signals may condition immune compartments beyond the involved lymph node [5]. Second, and most strikingly, Aoki and colleagues have recently published the first large-scale, multidimensional characterization of the tumor–immune architecture of cHL, integrating malignant-cell sequencing, spatial transcriptomics, and imaging mass cytometry to propose four molecular subtypes of the disease [6]—an important and promising advance that nevertheless remains an emerging, hypothesis-generating framework, not yet validated prospectively or ready to guide treatment, but with the potential to move the field from a unitary “immune-evasive lymphoma” concept toward a genuinely stratified disease model. Third, as summarized in recent reviews [7,8], clinical trials are now investigating whether circulating tumor DNA (ctDNA) and total metabolic tumor volume (TMTV) can guide treatment intensity; these strategies remain investigational and are not yet standard practice.

This review integrates these developments into a single, updated overview of cHL, organized around five questions that define what is genuinely new in the field: what is new in the biopathology of HRS cells and their microenvironment (Section 2); what is new in diagnosis, as pathology moves from morphology toward spatially resolved, artificial intelligence (AI)-assisted precision immunopathology (Section 3); what is new in the biopathology of the disease at the molecular level, including its emerging genomic taxonomy (Section 4); what is new in the understanding of cHL as a systemic disease and in how the newly described molecular subtypes connect to that systemic biology (Section 5 and Section 7); and what is new in prognosis, as tissue-based, circulating, and molecular biomarkers converge into integrated risk models now being explored in biomarker-directed clinical trials (Section 6).

### Review Methodology and Evidence Grading

This is a narrative, not a systematic, review. Literature was identified by the authors through searches of PubMed/MEDLINE up to mid-2026 using combinations of the terms “classic Hodgkin lymphoma”, “tumor microenvironment”, “single-cell”, “spatial transcriptomics”, “imaging mass cytometry”, “circulating tumor DNA”, “TARC/CCL17”, “molecular subtype”, and “clinical trial”, supplemented by the reference lists of key articles and by recent meeting abstracts (American Society of Hematology). Peer-reviewed full-length articles in English were prioritized; foundational earlier studies were included when they defined established concepts. No formal quality scoring or PRISMA-based selection was performed; so, selection bias cannot be excluded, and the inclusion of our own recent work [4,5] may reflect the authors’ perspective. Registered clinical trials were checked against ClinicalTrials.gov (accessed on 30 October 2026).

To help readers interpret the strength of the evidence, we use four descriptive categories throughout: (i) established practice (guideline-endorsed or routinely used in clinical decision-making); (ii) clinical observational evidence (retrospective or exploratory patient cohorts, associations without proof of causality); (iii) preclinical evidence (cell line, animal, or in vitro models); and (iv) hypotheses or conceptual models. Published full-length studies, meeting abstracts, and registered ongoing trials are explicitly distinguished in the text and in the tables. The molecular subtypes proposed by Aoki and colleagues [6] and the concept of cHL as a systemic immunobiological disease [5] are presented as emerging, hypothesis-generating frameworks, not as validated tools for staging or treatment selection.

## 2. Biopathology of HRS Cells and the Tumor Microenvironment

### 2.1. HRS Cells as Immune Modulators: Signaling and Genomic Drivers

HRS cells originate from germinal-center B cells that have largely lost their canonical B-cell transcriptional program through epigenetic reprogramming, yet retain clonal immunoglobulin gene rearrangements that unambiguously demonstrate their B-cell origin [1,9]. In place of a B-cell phenotype, HRS cells acquire activation markers, cytokine receptors, immune checkpoint ligands, and inflammatory mediators sustained by constitutive activation of two convergent pathways, NF-κB and JAK/STAT [9].

Constitutive NF-κB activation results from inactivating mutations of negative regulators such as TNFAIP3/A20 and NFKBIA and from chronic ligand-dependent stimulation through CD30, CD40, and RANK. In Epstein–Barr virus (EBV)-positive cHL, latent membrane protein 1 (LMP1) further amplifies NF-κB signaling by mimicking constitutively active CD40 while engaging JAK/STAT and AP-1 to increase PD-L1 expression independently of 9p24.1 [10,11]. JAK/STAT is independently dysregulated through recurrent alterations in SOCS1, STAT6, and PTPN1, with sustained STAT3/5/6 activation enhancing proliferation and cytokine production [9].

Amplification of chromosome 9p24.1—encompassing CD274 (PD-L1), PDCD1LG2 (PD-L2), and JAK2—is among the most clinically consequential lesions in cHL, simultaneously increasing checkpoint-ligand expression and JAK2-mediated signaling and providing the principal rationale for the efficacy of PD-1 blockade in newly diagnosed and relapsed/refractory disease [12,13,14,15].

HRS cells further remodel their environment through CCL17/TARC and CCL22, which recruit CCR4+ T-helper and regulatory T cells; IL-6, IL-10, and TGF-β, which sustain inflammation and suppress cytotoxic T-cell activity; and GM-CSF/M-CSF, which drive macrophage recruitment [3]. A less conventional mechanism, deletion of methylthioadenosine phosphorylase (MTAP), confers dependency on the PRMT5 arginine methyltransferase in several malignancies [16] and has recently been shown to remodel the immune landscape, with MTAP-deficient tumors displaying decreased T-/NK-cell infiltration and more immunosuppressive cells [17]; whether this operates similarly in cHL is under active investigation, with potential implications for PRMT5-directed therapeutics.

### 2.2. The Immune Cellular Ecosystem of cHL

The biological behavior of cHL is determined by reciprocal interactions between HRS cells and a heterogeneous network of immune and stromal cells rather than by any single dominant population (Table 1, Figure 1). CD4+ T-helper lymphocytes, recruited through CCL17/CCL22, are the principal cellular partner of HRS cells, providing survival signals through CD40/CD40L, ICOS/ICOSL, and CD2/CD58 that sustain NF-κB/JAK-STAT activation; these lymphocytes comprise multiple functional subsets whose activation and exhaustion state, rather than abundance, determine their contribution to disease progression [18,19].

Regulatory T cells (Tregs), recruited via CCL17/CCL22, suppress cytotoxic immunity through IL-10, TGF-β, and CTLA-4; their prognostic significance depends more on spatial localization relative to HRS cells than on absolute number [20]. CD8+ cytotoxic T cells are present but functionally impaired: HRS cells induce exhaustion through PD-L1 overexpression, defective antigen presentation, and chronic antigenic stimulation, and single-cell studies identify exhausted CD8+ populations co-expressing PD-1, TIGIT, LAG-3, and TIM-3 that concentrate around HRS cells and can be reinvigorated by PD-1 blockade [19].

Tumor-associated macrophages (TAMs), recruited through CCL2/CSF1, acquire immunoregulatory phenotypes marked by PD-L1, IL-10, and angiogenic-factor expression [21]; spatial transcriptomic studies show that macrophage localization within HRS-cell niches carries greater biological and prognostic significance than density alone [22]. NK cells are relatively scarce and functionally suppressed by TGF-β and inhibitory-receptor signaling [23], whereas eosinophils, mast cells, and cancer-associated fibroblasts contribute to matrix remodeling and chronic inflammation, particularly in nodular sclerosis, where fibrosis further limits immune-cell access to HRS cells [3].

### 2.3. Mechanisms of Immune Evasion

Immune evasion is a defining hallmark of cHL, allowing rare HRS cells to survive within an immunocompetent, highly inflammatory host. Escape results from the coordinated disruption of antigen presentation, activation of checkpoint pathways, induction of T-cell dysfunction, myeloid remodeling, and an immunosuppressive cytokine milieu, rather than any single mechanism.

The PD-1/PD-L1 axis remains the best-characterized escape mechanism [12,13]. Defective antigen presentation further limits antitumor immunity: MHC class I loss, commonly caused by B2M alterations, impairs CD8+ recognition [24], while CIITA gene fusions reduce MHC class II expression and compromise CD4+ activation [25]; preserved MHC class II expression has been associated with improved responses to PD-1 blockade [26].

A major conceptual advance is the characterization of T-cell exhaustion as a continuum rather than a binary state. Progenitor exhausted T cells (Tpex), marked by TCF1 and retained self-renewal capacity, are the reservoir from which effector responses regenerate; transitory exhausted cells are the primary responders to PD-1 blockade [27]; and terminally exhausted cells, marked by TOX, TIM-3, and TIGIT, are largely refractory to reinvigoration owing to fixed epigenetic silencing [28,29]—a framework with direct implications for predicting checkpoint responsiveness.

The immunosuppressive cytokine milieu reinforces escape: IL-10 and TGF-β suppress antigen presentation and cytotoxic activity, while IL-6 sustains JAK/STAT signaling; additional mediators, including galectin-1, further foster immune privilege and provide potential targets for combination immunotherapy [30]. Finally, immune suppression in cHL is spatially organized rather than uniformly distributed: topological analyses show that physical proximity between PD-L1-expressing niches and HRS cells, more than immune-cell abundance, shapes function [31], positioning spatial architecture as a biomarker explored further in Section 3, Section 4, Section 5 and Section 6.

## 3. Diagnosis: From Morphology to Precision Immunopathology

### 3.1. Histopathological and Immunophenotypic Biomarkers

Histopathological examination remains the cornerstone of cHL diagnosis, integrating morphology with immunophenotyping of HRS cells within their characteristic inflammatory background according to the WHO 5th edition and International Consensus Classification frameworks [32,33,34]. HRS cells typically express CD30, CD15 (~75–85% of cases), MUM1/IRF4, and weak PAX5, while usually lacking CD20, CD79a, and CD45; although highly reproducible, this immunophenotype provides limited insight into the functional state of the immune microenvironment.

Among emerging biomarkers, PD-L1 is the most clinically relevant: although not required for routine diagnosis, PD-L1 immunohistochemistry may assist in challenging cases and links pathology directly to immunotherapy, being expressed by both HRS cells and TAMs [13]. MHC assessment provides complementary information: MHC class I loss, usually via B2M alterations, impairs CD8+ recognition [24], while reduced MHC class II, often via CIITA dysregulation, limits CD4+ activation [25]. EBER in situ hybridization remains the gold standard for EBV-associated cHL, a subtype with distinct PD-L1 expression, cytokine profiles, and macrophage/T-cell composition [11,35].

### 3.2. Spatial Organization of the Tumor Microenvironment

The spatial organization of the TME has emerged as a fundamental determinant of cHL biology and a genuinely new frontier for diagnostic pathology: the physical proximity, neighborhoods, and functional interactions between HRS cells and surrounding immune cells provide diagnostic, prognostic, and predictive information beyond cellular composition alone [22,31]. Multiplex immunohistochemistry/immunofluorescence, imaging mass cytometry, digital spatial profiling, and spatial transcriptomics now allow for simultaneous characterization of multiple populations while preserving tissue architecture [20,36,37,38].

T cells and macrophages adjacent to HRS cells display transcriptional programs distinct from phenotypically similar cells elsewhere in the tissue, indicating that local microenvironmental signals continuously shape immune-cell behavior. HRS cells occupy specialized niches enriched in exhausted PD-1+ T cells, FOXP3+ Tregs, PD-L1+ macrophages, and activated fibroblasts, providing a mechanistic explanation for the sensitivity of cHL to PD-1 blockade. Digital pathology and AI now enable automated HRS-cell identification and reconstruction of cellular interaction networks from routine tissue sections [39,40,41], though high-dimensional platforms remain technically demanding, and variability in processing and analytical pipelines currently limits inter-study comparability.

## 4. What Is New in Biopathology: Molecular Subtyping of cHL

For decades, cHL has resisted the comprehensive molecular classification that has transformed diffuse large B-cell lymphoma and other aggressive lymphomas, chiefly because HRS cells constitute only 1–10% of tumor cellularity, leading bulk genomic assays to be dominated by a non-neoplastic signal. Two complementary strategies have begun to overcome this obstacle. The first relies on ctDNA to non-invasively capture HRS-cell genetics from plasma; using this approach, Alig and colleagues recently defined distinct genomic subtypes of Hodgkin lymphoma from noninvasive profiling (an H1/H2 classification), establishing proof of concept that HRS-cell genomics can be resolved without tissue microdissection [42]. These two classifications were derived using different sample sources and analytical approaches, and they are compared in Table 2 and Table 3. The second, more comprehensive strategy directly integrates malignant-cell-enriched sequencing with spatial technologies applied to the intact tumor architecture.

In 2026, Aoki and colleagues reported the largest and most comprehensive application of this second strategy to date [6]. Using laser-capture microdissection and fluorescence-activated cell sorting to enrich HRS cells from 114 frozen diagnostic biopsies (103 treated with ABVD or ABVD-equivalent regimens), they performed whole-exome and targeted capture sequencing, non-negative matrix factorization (NMF) clustering on 80 recurrently altered genes/loci, GeoMx digital spatial transcriptome profiling of CD30+ HRS cells, and imaging mass cytometry of the intact microenvironment. This represents, in our view, an important and promising biopathological development: it links specific genomic drivers of HRS cells to reproducible, spatially defined immune configurations and distinct clinical phenotypes (Table 2). Nevertheless, this is a single-study, hypothesis-generating framework that has not yet been independently replicated or validated prospectively, and it should not be regarded as an established tool for treatment selection. No significant correlation was found between the resulting molecular subtypes and conventional histologic subtype, indicating that this taxonomy captures a biological axis orthogonal to standard morphology.

Four molecular subtypes emerged, termed CST (31% of the cohort), CN913 (25%), STB (25%), and CN2P (18%). CST is defined by heterozygous, predominantly truncating mutations affecting the cytoplasmic domain of CSF2RB (24.3% of an extended in-house cohort of 107 cases, with a recurrent E788* hotspot), frequently co-occurring with TNFAIP3, MFHAS1, GNA13, and BCL7A alterations; functional modeling in cell-line systems indicated that these are gain-of-function, driving ligand-dependent hyperphosphorylation of STAT5 downstream of CSF2RB–JAK2. The CST microenvironment is enriched for FOXP3+ Tregs and CCR4+ T cells, and CST is the only subtype in which TARC/CCL17+ HRS cells show significant spatial interaction with CCR4+CD4+ T cells, consistent with a mutation-driven TARC–CCR4 axis recruiting Tregs into the tumor niche; CST is preferentially observed in younger patients.

CN913 is defined by copy-number gain of 9p24.1 (JAK2, CD274/PD-L1, PDCD1LG2/PD-L2) together with 13q deletion, plus 6q23.3/1p36.32 deletions and KMT2D/TP53/KMT2A alterations. Its microenvironment is enriched for LAG-3+CD4+ T cells and PD-L1+ macrophages clustered around HRS cells, with prominent interferon-γ and NF-κB upregulation. CN913 is strongly associated with EBV positivity (~79% of cases; OR 6.89), independently confirmed in an external validation cohort.

STB is defined by mutations in STAT6 and B2M (plus SOCS1, XPO1, IKBKB, EEF1A1), with B2M mutations driving MHC class I loss. Its microenvironment is immune-cold, with fewer Granzyme B+ CD8+ T cells and upregulation of a TGF-β signature in HRS cells; a distinctive galectin-9+ HRS–TIM-3+ macrophage interaction was identified specifically in this subtype. Although no significant progression-free survival (PFS) differences were seen across subtypes in the discovery cohort, STB showed a trend toward inferior PFS, consistent with its disproportionate representation among relapsed biopsies (4/10, 40%)—hypothesis-generating and requiring confirmation in larger, prospective cohorts. CN2P is characterized by gain of 2p15 (REL, BCL11A) and 12q13.3, with upregulated DNA-repair/TP53-target signatures and recurrent NFKBIA, IL4R, EGR1, and FAS alterations; its microenvironment is enriched for PD-1+CD4+ T cells near HRS cells, with a specific PD-L1+HRS–PD-1+CD4+ interaction consistent with an exhausted phenotype. CN2P preferentially affects older patients (validation cohort, *p* < 0.0001).

Beyond their descriptive value, Aoki and colleagues translated the CST-defining CSF2RB mutations into a proof-of-concept therapeutic hypothesis: in isogenic cell-line models and a humanized xenograft, mutant CSF2RB constitutively activated STAT5 in a cytokine (IL-3/IL-5)-dependent manner, increased TARC/CCL17 and IL-13 production, promoted CCR4+ T-cell (including Treg) migration and splenic Treg infiltration in vivo, and these gain-of-function phenotypes were reversed by the selective JAK2 inhibitor pacritinib—an experimental (preclinical) rationale for JAK2 inhibition in the CST subtype; the clinical activity of pacritinib in cHL has not been demonstrated. More broadly, the four subtypes generate additional testable hypotheses about differential therapeutic sensitivity: the PD-L1/PD-L2-high, EBV- and interferon-enriched CN913 subtype might derive particular benefit from PD-1 blockade, whereas the MHC class I-deficient, immune-cold STB subtype might be less responsive to checkpoint inhibition alone. Even the CSF2RB/JAK2 axis is supported only by preclinical data, and the remaining hypotheses are biologically plausible but not clinically established; the companion perspective by Aoki and Kuruvilla explicitly frames the opportunity to align this biological stratification with treatment-intensity decisions, particularly in early-stage, favorable-risk disease [43].

To enable application of this taxonomy beyond the discovery cohort, the authors developed and publicly released HLGen, an ensemble classifier trained on the 80 NMF-defining features that assigns patients to one of the four subtypes (or “UNCLASS” when confidence is low) from binary mutation/copy-number data alone. HLGen was validated in an independent cohort of 293 patients profiled by ctDNA-based targeted sequencing—the same cohort used by Alig and colleagues [42]—reproducing all four classes and confirming the CN913–EBV and CN2P–age associations, and is compatible with reduced gene panels used in noninvasive ctDNA testing, potentially bridging tissue-based discovery with the liquid biopsy strategies discussed in Section 6. Because each subgroup carries a distinct immune coinhibitory-receptor profile (PD-L1, PD-1, LAG-3), the authors propose HLGen as a candidate predictive-biomarker framework for therapeutic target selection, pending prospective validation. HLGen is a research classifier: it has not been prospectively validated, is not a clinical-grade assay, and should not currently be used to guide therapy.

Alternative and complementary classifications. The framework of Aoki and colleagues is not the only genomic taxonomy proposed for cHL. The ctDNA-based H1/H2 classification of Alig and colleagues [42] was derived from plasma profiling of 366 patients, whereas the four HLGen classes were derived from HRS-cell-enriched tissue and then re-examined in ctDNA from an independent cohort [6] (Table 3). The two approaches differ in sample source, genomic inputs, number of classes, and the additional layers of data (spatial transcriptomics and imaging mass cytometry in the tissue-based study); to our knowledge, the concordance between H1/H2 and the HLGen classes has not been formally reported; so, the two schemes cannot yet be considered interchangeable. Both remain research-level classifications: neither has been validated prospectively, neither is used to assign therapy, and, at present, histologic subtype according to WHO/ICC criteria [32,33,34], EBV status, and 9p24.1/PD-L1 biology remain the only established biological descriptors in routine practice.

Taken together, this new taxonomy complements—rather than replaces—the immune-evasion framework described in Section 2: the genomic lesions driving NF-κB/JAK-STAT activation, 9p24.1 amplification, and antigen-presentation loss are not randomly distributed across cHL but cluster into reproducible tumor–immune ecosystems with distinct clinical correlates. Whether these ecosystems also leave a detectable footprint beyond the lymph node—in the peripheral immunological and cytokine/chemokine profile that defines cHL as a systemic disease—is examined in Section 7.

## 5. cHL as a Systemic Immunobiological Disease

Classic Hodgkin lymphoma has long been conceptualized as a malignancy of the lymphatic system with orderly, contiguous nodal spread—a framework formalized in staging systems such as Ann Arbor and Lugano that has guided management for decades. As we have recently argued, however, this anatomical framework provides an incomplete representation of cHL biology and does not fully account for several clinical and molecular features of the disease [5]. We stress that this is a proposed conceptual model: it is intended to complement, not replace, anatomical staging (Ann Arbor/Lugano), which remains the standard for staging and treatment allocation.

Accumulating, largely observational, evidence suggests that HRS-cell–driven interactions extend well beyond the lymph node. Circulating tumor DNA [44], soluble mediators such as TARC/CCL17, and systemic alterations in peripheral immune compartments are compatible with a model of disease-driven, body-wide immune reprogramming (Figure 2), although direct proof that HRS cells cause these peripheral changes is limited. Peripheral blood analyses in patient cohorts have reported functional exhaustion of circulating T cells, including PD-1 upregulation and impaired effector responses that are detectable outside the tumor-infiltrating compartment, together with expansion of regulatory T cells that contributes to a suppressive systemic immune tone [45,46]. The CST-subtype TARC–Treg axis (Section 7) is the only mechanistic link between a specific genomic lesion and a systemic readout explored experimentally to date, and its peripheral component has been shown only in a xenograft model.

The myeloid compartment is similarly altered, with increased circulating monocytes bearing immunosuppressive features and signatures consistent with myeloid-derived suppressor cells, associated with adverse outcomes [47]. These abnormalities are dynamic: restoration of immune competence following effective therapy, particularly checkpoint blockade, suggests their functional relevance and potential reversibility, although incomplete normalization in some patients suggests persistent dysregulation may contribute to resistance and relapse.

This systemic dimension is clinically reflected in constitutional (“B”) symptoms—fever, night sweats, weight loss—putatively linked to cytokine production (causality not established), with circulating IL-6 and TARC/CCL17 correlating with disease activity. Extranodal involvement (bone marrow, liver, lung) is another key correlate, reflecting dissemination beyond lymphoid tissue and more advanced, distinct clinical behavior [48,49]; these sites may be not merely passive locations of spread but biologically active niches shaped by tumor-derived signals and host immune responses, possibly recapitulating nodal microenvironment features, an interpretation that remains largely inferential (Table 4) [50].

Treatment response may be determined not solely by tumor burden but also by the state of the immune microenvironment and systemic immune competence, and resistance to checkpoint blockade may reflect persistent T-cell dysfunction and compensatory immunosuppressive pathways rather than intrinsic tumor resistance alone. Reframing cHL as a systemic immunobiological disease—a proposed model whose link to molecular subtypes has so far been tested only in part (Section 7)—may have implications for disease assessment (Section 3) and for risk stratification and biomarker-directed monitoring (Section 6), and would support exploring the integration of molecular and immune biomarkers alongside, not instead of, anatomical staging, pending prospective validation.

Strength of evidence. In this section, the following elements are supported by observational patient data: circulating ctDNA and TARC/CCL17 correlating with disease activity [44]; T-cell exhaustion and Treg expansion in peripheral blood [45,46]; circulating myeloid-derived suppressor cells associated with outcome [47]; and extranodal involvement [48,49,50]. In contrast, the causal contribution of HRS-derived mediators to these peripheral changes, the role of extracellular vesicles and soluble PD-L1, and the concept of extranodal sites as tumor-conditioned niches are inferential or hypothetical. Preclinical evidence is limited to the CSF2RB-mutant xenograft model discussed in Section 7.

## 6. Prognosis: Tissue, Circulating, and Integrated Biomarkers

### 6.1. Traditional Clinical Prognostic Factors

Traditional factors—disease stage, bulky disease, erythrocyte sedimentation rate, and interim PET—remain fundamental to clinical decision-making in early-stage cHL, while the International Prognostic Score (IPS) [51] and the newer Advanced-stage Hodgkin Lymphoma International Prognostic Index (A-HIPI) [52] retain significance with modern regimens, though they are not used to directly guide therapy [53,54]. These parameters primarily reflect disease burden rather than the biology underlying treatment response; prognostic assessment in cHL is therefore progressively evolving from clinical risk stratification toward integrated biological profiling (Table 5).

Established versus investigational biomarkers. Currently used in routine decision-making are disease stage, bulky disease, B symptoms, erythrocyte sedimentation rate, and interim/end-of-treatment PET-CT (Deauville criteria), the latter guiding response-adapted therapy [54]; IPS and A-HIPI are prognostic scores not used to direct therapy. By contrast, tissue immune and spatial biomarkers, TARC/CCL17 and other cytokines, blood cell ratios, ctDNA, and the molecular subtypes/HLGen are investigational and are discussed below as such.

### 6.2. Tissue-Based Immune Biomarkers

Among tissue biomarkers, TAMs remain the most extensively investigated: increased CD68+ macrophages were initially linked to shorter survival [21], though subsequent studies produced conflicting results. Spatial and single-cell analyses have refined this interpretation, showing that immune-cell positioning frequently carries more prognostic information than absolute numbers, with immunosuppressive PD-L1+CD163+IDO1+ macrophages preferentially accumulating in direct contact with HRS cells [22,38].

PD-L1 expression is another widely studied biomarker: although constitutive overexpression characterizes nearly all cHL, quantitative differences, 9p24.1 amplification, and distribution of PD-L1+ immune cells have been associated with distinct outcomes [12,13]. PD-L1 should be interpreted primarily as predictive of checkpoint-inhibitor benefit rather than purely prognostic. Preservation of MHC class II expression has similarly been associated with superior outcomes after PD-1 blockade, likely because effective CD4+ T-cell activation remains possible despite checkpoint-mediated suppression [25,26].

### 6.3. Circulating Immune and Inflammatory Biomarkers

Circulating biomarkers are attractive because blood-based assays are minimally invasive and suitable for longitudinal monitoring. TARC/CCL17 is, among circulating markers, supported by the most extensive evidence: serum concentrations correlate with tumor burden and PET metabolic activity, and rapid normalization during treatment has been associated with favorable response, whereas persistent elevation has been associated with residual disease or relapse [55,56]. Because TARC is elevated in most newly diagnosed patients while remaining normal in healthy individuals, it is a promising investigational biomarker for diagnosis, monitoring, and residual disease assessment [57]. However, its use is limited by the lack of assay standardization (different assay platforms and sample matrices, serum versus plasma), the absence of universally accepted cutoffs, reduced specificity (elevation has been described in non-lymphomatous inflammatory conditions, such as atopic skin disease, and in other lymphomas), and the lack of prospective trials showing that TARC-guided management improves outcomes [55,56].

Additional cytokines—IL-6, IL-10, TNF-α, soluble CD30, CXCL13—have also been investigated, with elevated levels generally reflecting increased inflammatory activity and tumor–host interaction, though routine implementation remains limited by biological variability [58]. Routine hematological indices have gained attention for their availability: elevated neutrophil-to-lymphocyte ratio has been associated with inferior progression-free survival across retrospective studies [59,60], and additional indices including lymphocyte-to-monocyte, platelet-to-lymphocyte, and systemic immune-inflammation ratios show similar associations [61]. Analysis of ctDNA is a promising investigational tool: in retrospective or exploratory series, rapid clearance after treatment has been associated with durable remission, whereas persistent molecular disease has been associated with relapse risk, in some cases before radiological progression [44,62]. Limitations include the lack of standardized assays and MRD definitions (different platforms, sampling time points, and thresholds), variable sensitivity depending on tumor burden and HRS-cell DNA shedding, and the absence, so far, of randomized evidence that ctDNA-guided treatment modification improves outcomes (Table 5). Neither TARC nor ctDNA is currently incorporated into guideline-based response assessment, which remains imaging-based.

### 6.4. Spatial and Molecular Prognostic Biomarkers: Linking Prognosis to Molecular Subtypes

Spatial immune architecture has been reported to be an independent prognostic biomarker in relapsed/refractory cHL beyond conventional immune-cell quantification [38,63], but these findings derive from retrospective cohorts and await prospective validation and assay standardization. The molecular subtypes described in Section 4 add a complementary layer: no significant PFS difference was observed across the four HLGen subtypes in the discovery cohort, but the immune-cold, MHC class I-deficient STB subtype showed a trend toward inferior PFS, consistent with its disproportionate representation among relapsed biopsies (40%)—a hypothesis-generating signal requiring confirmation in larger, prospective cohorts [6]. Each subtype also generates testable predictive hypotheses (the immune-cold STB subtype expected to respond less favorably to checkpoint inhibition than the interferon-activated CN913 subtype, for example), and the distinct coinhibitory-receptor profile of each HLGen subgroup has led the original authors to propose molecular subtyping as a candidate predictive-biomarker framework, complementing the peripheral cytokine correlates discussed in Section 7 [6].

AI is a further rapidly evolving field: machine-learning algorithms can quantify immune-cell composition and spatial organization and integrate histological with molecular and clinical variables, offering reproducible prognostic models that may eventually reduce interobserver variability [39,40,41]. HLGen itself, the classifier developed alongside the Aoki subtypes, illustrates how computational classification and biomarker discovery are converging, although HLGen is currently a research tool rather than validated clinical-decision support. The prognostic landscape of cHL may thus be shifting from conventional clinicopathological scoring toward biologically informed precision medicine; the greatest impact will likely come not from individual biomarkers but from integrated models combining immune, molecular, pathological, and clinical information to guide personalized therapy (Table 5).

**Table 5:** Integrated biomarker framework (conceptual) and investigational biomarker-directed clinical trials in classic Hodgkin lymphoma.

Domain	Representative Biomarkers/Trials	Clinical Application
Baseline biological risk stratification	IPS/A-HIPI + tissue immune biomarkers + TARC + ctDNA + inflammatory indices + molecular subtype (conceptual framework proposed by the authors; not validated)	Single integrated biological profile rather than separate risk estimates
ctDNA-directed MRD assessment	GHSG HD21 (LymphoVista MRD-2 assay) [64]—peer-reviewed retrospective case–cohort analysis	4-year PFS 36.7% (MRD-2+) vs. 82.2% (MRD-2−); combined with interim PET-2; prognostic only, no ctDNA-guided intervention tested
ctDNA-guided de-escalation (frontline)	SWOG S1826 [65] ctDNA substudy [66]; PRECISE-HL (NCT06745076)—ctDNA substudy reported as a meeting abstract; PRECISE-HL is a phase II trial, recruiting (primary endpoint: PFS in patients with undetectable MRD after 2 cycles)	Undetectable ctDNA after 2 cycles nivo-AVD → 4 cycles of nivo-AVD (followed by nivolumab alone) versus 6 cycles of nivo-AVD if ctDNA remains detectable (investigational)
ctDNA/PET-guided consolidation (R/R disease)	UC Hematologic Malignancies Consortium pembro-GVD (NCT07021989) [67]—meeting abstract; phase II trial, recruiting	Response-adapted (PET and ctDNA) pembrolizumab + GVD; selected patients may receive non-transplant consolidation (pembrolizumab and/or 30 Gy ISRT) instead of HDT/ASCT (investigational; primary endpoint: ctDNA/MRD-negative, PET-negative complete response rate)
TMTV-directed therapy	NCT04866654 (early-stage non-bulky); NCT06377566 (early-stage bulky) [68]—both phase II: NCT04866654 (RAFTING; registry status “unknown”, last updated 2021) and NCT06377566 (BV-AVD; recruiting); [68] provides general background	Tumor-burden-adapted treatment intensity (investigational; primary endpoints: 3-year PFS and PFS, respectively)
Longitudinal surveillance	Serial ctDNA, TARC, circulating immune signatures, AI-assisted analysis [69] (exploratory; supported by abstract-level data only)	Early relapse detection; reduced reliance on repeated imaging (investigational)

ASCT, autologous stem-cell transplantation; ctDNA, circulating tumor DNA; GVD, gemcitabine/vinorelbine/liposomal doxorubicin; nivo-AVD, nivolumab plus doxorubicin/vinblastine/dacarbazine; TMTV, total metabolic tumor volume. HRS, Hodgkin and Reed–Sternberg; ISRT, involved-site radiotherapy; MRD, minimal residual disease; PFS, progression-free survival. All approaches listed are investigational and none represents standard practice. Trial data are those reported in ClinicalTrials.gov (accessed on 30 October 2026): NCT06745076 (phase II, recruiting), NCT07021989 (phase II, recruiting), NCT06377566 (phase II, recruiting), NCT04866654 (phase II, registry status “unknown”). References [66,67,69] are meeting abstracts; Ref. [64] is a peer-reviewed retrospective case–cohort analysis; Ref. [65] is the published randomized phase 3 trial, without a ctDNA-guided intervention.

## 7. Bridging Molecular Subtypes and Systemic Immunobiology

A key question raised by this new taxonomy is whether subtype-defining genomic lesions leave a detectable footprint beyond the lymph node—that is, whether molecular biopathology (above) and the systemic immunobiological model of cHL (Section 5) describe the same underlying process (Figure 3) at different scales. The clearest evidence comes from the CST subtype. Because mutant CSF2RB drives ligand-dependent STAT5 activation and increased TARC/CCL17 and IL-13 secretion, Aoki and colleagues showed that this genotype–phenotype link is not confined to the tumor niche: CSF2RB-mutated cases displayed significantly higher CCL17 transcript levels and, by immunohistochemistry, no TARC-negative cases at all (versus a TARC-negative subset among CSF2RB-wild-type cases), and in the xenograft model, mice bearing CSF2RB-mutant tumors showed significantly elevated serum TARC together with increased splenic CD4+CD25+FOXP3+ Treg infiltration relative to wild-type controls. This provides a genotype-anchored mechanistic link between a specific molecular subtype and the circulating TARC biomarker investigated for diagnosis and monitoring (Section 6) and the systemic Treg-driven immune reprogramming described in Section 5, and it is an example in cHL in which a discrete genomic lesion has been linked experimentally from HRS-cell signaling to a peripheral cytokine and immune-cell phenotype. It should be noted, however, that the peripheral component (serum TARC and splenic Tregs) was demonstrated only in a xenograft model, whereas in patients the evidence is limited to tissue-level CCL17 expression and TARC immunohistochemistry; confirmation of the serum readout in patients is still pending.

The remaining subtypes suggest testable, but as-yet largely unproven, systemic correlates. The EBV-enriched, interferon/NF-κB-driven CN913 subtype would be expected, on mechanistic grounds, to associate with a systemic interferon-skewed inflammatory profile and elevated circulating inflammatory indices, paralleling the known systemic cytokine signature of EBV-associated cHL; the immune-cold, TGF-β-high STB subtype might instead correspond to a comparatively blunted peripheral inflammatory and cytokine response; and the PD-1-driven exhaustion niche that defines CN2P plausibly extends to a circulating PD-1hi T-cell exhaustion phenotype similar to that already documented systemically in cHL. None of these three hypotheses has yet been directly tested by paired tissue–blood profiling, and they are presented here as a research agenda (Section 8; Figure 3) rather than as established findings. Taken together, however, the CST–TARC–Treg axis demonstrates in principle that Aoki’s molecular subtypes are not confined to a static, tissue-only classification: they have the potential to be read out, at least in part, from the peripheral immunological and cytokine/chemokine profile that defines cHL as a systemic disease.

## 8. Future Directions

Recent advances have established the TME as a central determinant of the biology, clinical behavior, and therapeutic response of cHL, and the 2026 description of candidate molecular subtypes may mark a further step toward a more biology-driven disease model, provided that it is validated prospectively. Translating these discoveries into practice nonetheless remains challenging, as few biomarkers have achieved the reproducibility needed for widespread implementation given differences in cohorts, protocols, tissue processing, and analytical platforms.

An emerging priority is spatial characterization of the TME beyond bulk immune-cell quantification: multiplexed platforms including imaging mass cytometry, CODEX, and spatial transcriptomics increasingly indicate that the proximity, directionality, and functional state of immune-cell interactions—rather than aggregate abundance—may determine immune exclusion and checkpoint resistance [38,63]; the CODEX platform itself was originally described in mouse spleen [70]. In cHL, niche-level analyses suggest the existence of microanatomical structures that may act as reservoirs of progenitor-exhausted (Tpex) T cells capable of mounting a productive checkpoint response, and the spatial relationship between HRS cells, PD-1+ T cells, and macrophage subsets may influence the probability of T-cell reinvigoration after PD-1 blockade.

A major limitation is that most biomarker studies, including the Aoki molecular subtyping study, rely on single diagnostic biopsies, providing only a static view of a highly dynamic ecosystem. Prospective validation of the CST, CN913, STB, and CN2P subtypes in independent cohorts, and their incorporation as stratification factors in future trials, is a clear near-term priority; equally important are longitudinal, paired tissue-and-blood studies that directly test the systemic cytokine and immune correlates proposed in Section 7 for the CN913, STB, and CN2P subtypes, and that determine whether molecular subtype itself remains stable or shifts under treatment pressure.

Integrating ctDNA dynamics with TME profiling and molecular subtyping is among the most promising near-term translational opportunities; because ctDNA reflects tumor burden and clonal dynamics rather than immune context directly, parallel profiling of immune cell-derived cell-free fragments, peripheral T-cell receptor diversity, and soluble checkpoint molecules alongside ctDNA may generate composite liquid biopsy signatures capturing both tumor-intrinsic and immune-extrinsic resistance mechanisms [71]. Post-transplant immune reconstitution after prior checkpoint-inhibitor exposure is a further unmet need at the intersection of TME biology and transplant medicine, where prospective studies correlating pre-transplant immune phenotype and molecular subtype with outcome would help inform patient selection; the TME-based rationale for targeting primary refractory and early-relapsed disease has been reviewed previously [72].

Taken together, the priorities for the field are to prospectively validate the molecular subtypes and their proposed systemic correlates; to develop and validate integrated multidimensional models combining tissue, circulating, spatial, and molecular data; to standardize spatial and digital pathology platforms and AI-based image analysis through international collaboration; to characterize longitudinally how the immune microenvironment and molecular subtype evolve during treatment and at relapse; and to translate systems immunology into precision medicine through prospective, biomarker-stratified multicenter trials. AI is becoming essential to this effort: deep-learning algorithms already identify HRS cells, quantify immune populations, and integrate morphological, genomic, and clinical data, and explainable, interpretable AI—supported by large, annotated, multicenter repositories—is expected to support, rather than replace, expert hematopathologists.

Limitations of this review: This is a narrative review with a non-systematic literature search; selection bias cannot be excluded. The Aoki et al. framework rests largely on a single discovery study with a limited sample size and without prospective clinical validation, and several of the biomarkers discussed are supported only by retrospective data, meeting abstracts, or ongoing trials whose results are not yet available. Because the field is evolving rapidly, some conclusions may change as new data are published.

## 9. Conclusions

Classic Hodgkin lymphoma is being reframed along three complementary axes. Biologically, it may be emerging as a molecularly stratified disease—as suggested by the CST, CN913, STB, and CN2P subtypes proposed by Aoki and colleagues, a framework that still requires validation—rather than a single, uniform immune-evasive entity. Clinically, it has been proposed to be a systemic immunobiological disorder in which HRS-derived circulating mediators may condition immune compartments and extranodal niches well beyond the involved lymph node, as illustrated experimentally by the CST-subtype TARC–Treg axis (Section 7), a model intended to complement, not replace, anatomical staging. And diagnostically and prognostically, cHL assessment is gradually moving from morphology- and stage-only evaluation toward integrated precision immunopathology combining histopathology, immunophenotyping, spatial biology, liquid biopsy, and computational analysis.

None of these advances alone is yet sufficient to replace established practice. Histopathological evaluation, integrating morphology, immunophenotyping, and, in selected cases, molecular analysis under WHO 5th edition/ICC criteria, remains the diagnostic gold standard, and PD-L1 overexpression—driven chiefly by 9p24.1 amplification and, in EBV-positive cases, LMP1 signaling—remains the central biological rationale for PD-1 inhibitor therapy. Considerable work remains to validate the molecular subtypes and their systemic correlates prospectively, to standardize spatial and liquid biopsy assays, and to demonstrate that biomarker-guided treatment adaptation improves outcomes in randomized trials; no single biomarker, whether clinical, tissue-based, circulating, spatial, or molecular, is yet sufficient on its own for routine diagnostic or prognostic use. Circulating biomarkers, particularly TARC/CCL17 and ctDNA, are nonetheless promising investigational tools for disease monitoring and minimal residual disease assessment and are being evaluated for biomarker-directed treatment adaptation in ongoing trials, but they are not yet part of routine clinical decision-making.

Together, the biological stratification introduced by multidimensional tumor–immune architecture profiling, its emerging links to systemic immunobiology, the proposed reconceptualization of cHL as a systemic disease, and the evolving biomarker-directed clinical trial landscape may chart a path toward integrated, biology-driven, personalized management of classic Hodgkin lymphoma—the model that gives this review its title.

## Figures and Tables

**Figure 1:**
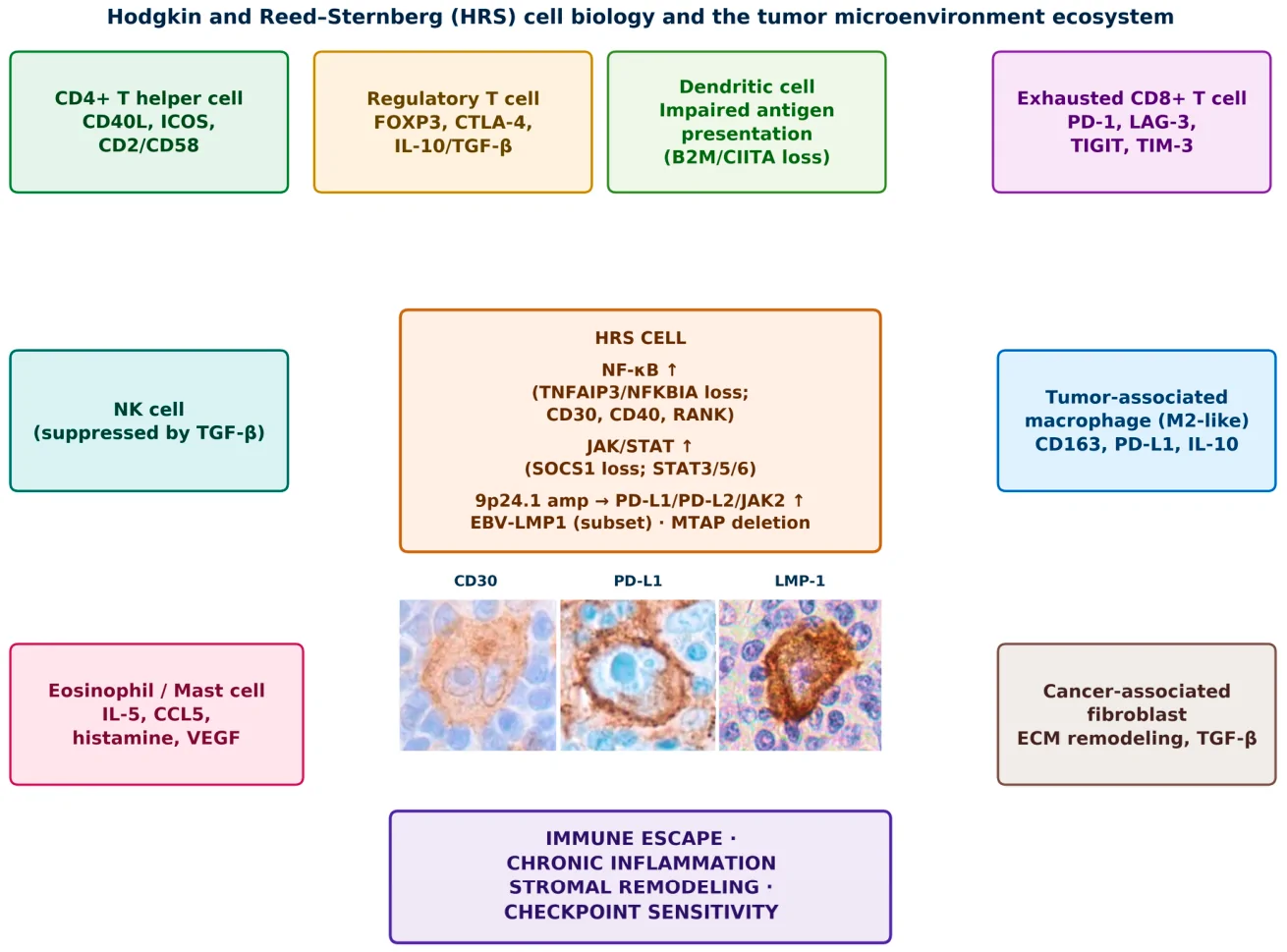
Hodgkin and Reed–Sternberg (HRS) cell biology and the tumor microenvironment ecosystem. HRS cells sustain constitutive NF-κB and JAK/STAT signaling and 9p24.1-driven PD-L1/PD-L2 overexpression, and secrete chemokines and cytokines that recruit and functionally reprogram CD4+ T cells, regulatory T cells, exhausted CD8+ T cells, tumor-associated macrophages, NK cells, eosinophils/mast cells, and cancer-associated fibroblasts, collectively sustaining immune escape, chronic inflammation, stromal remodeling, and checkpoint sensitivity. The schematic is conceptual. Representative immunohistochemical staining for CD30, PD-L1, and LMP1 is shown in the lower panels (classic Hodgkin lymphoma, EBV positive, original magnification 40×.

**Figure 2:**
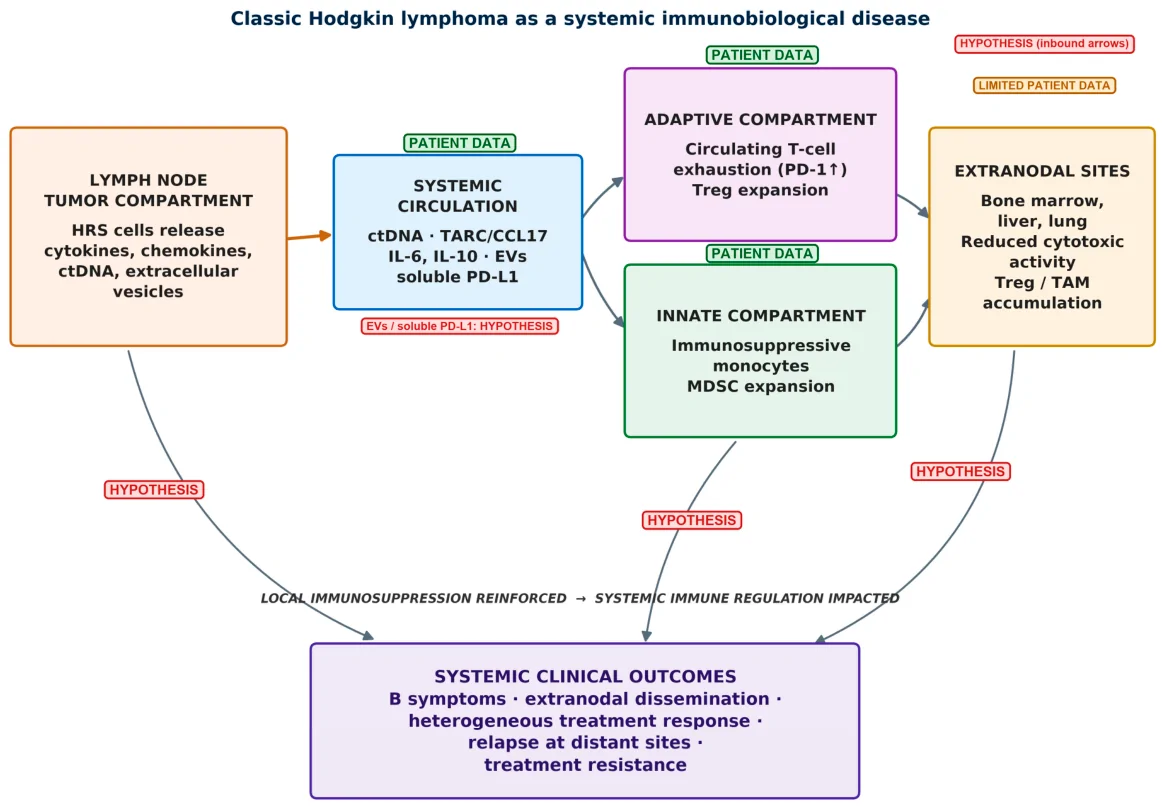
Classic Hodgkin lymphoma as a systemic immunobiological disease. HRS cells release cytokines, chemokines, circulating tumor DNA (ctDNA), and extracellular vesicles into the systemic circulation, driving coordinated dysregulation of adaptive (T-cell exhaustion, Treg expansion) and innate (immunosuppressive monocytes, MDSC expansion) immune compartments and conditioning extranodal sites (bone marrow, liver, lung), with downstream systemic clinical consequences. Evidence level: green “patient data” labels indicate elements supported by observational studies in patients [44,45,46,47]; red “hypothesis” labels indicate inferred or unproven links (including soluble PD-L1/extracellular vesicles, the convergence on extranodal sites, and the causal link to clinical outcomes). The figure is conceptual and does not imply proven causality.

**Figure 3:**
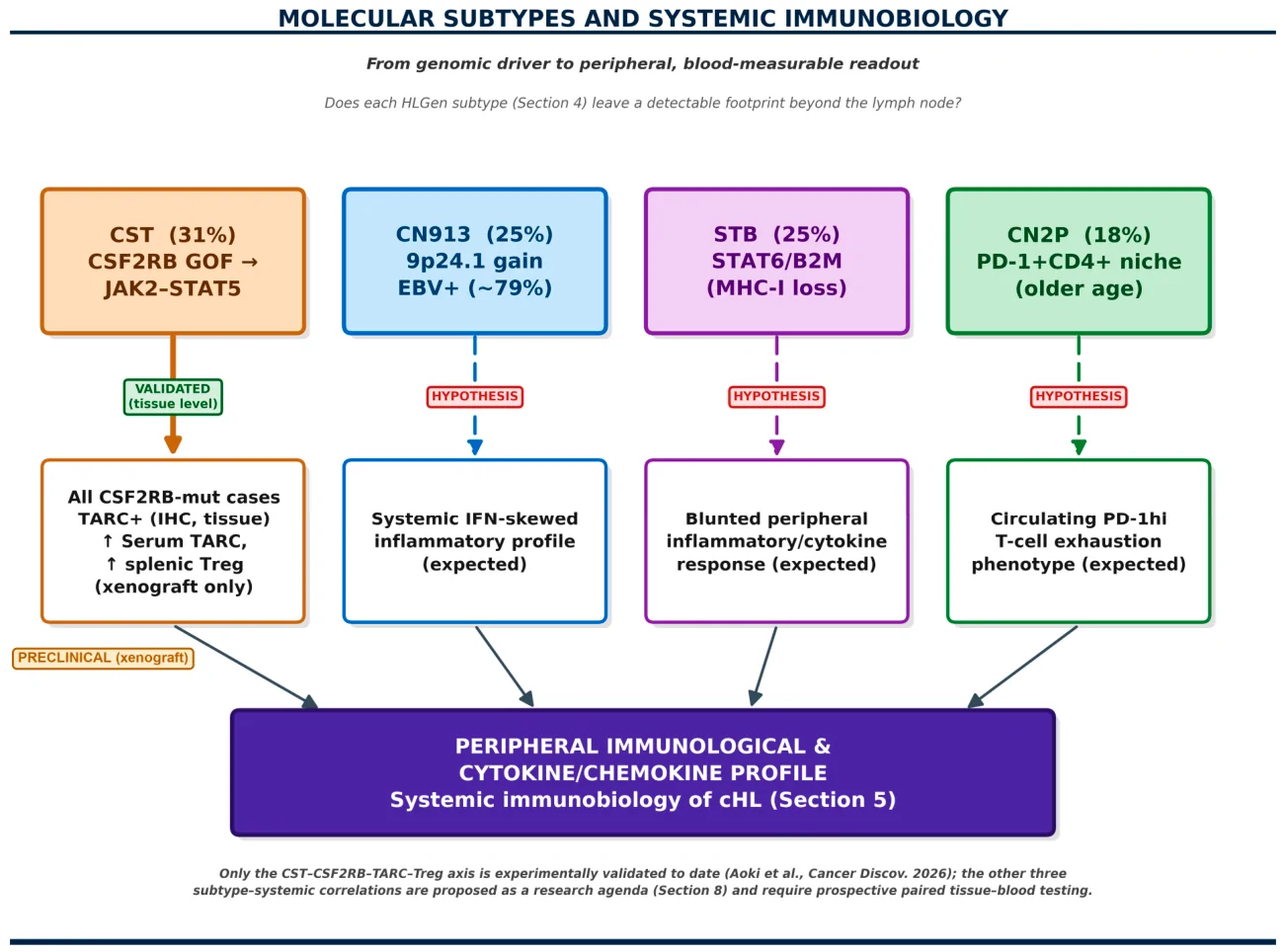
Molecular subtypes and systemic immunobiology: from genomic driver to peripheral readout. The CST subtype’s CSF2RB–JAK2–STAT5–TARC/CCL17–Treg axis is validated at the tissue level in patients (CCL17 transcript and TARC immunohistochemistry) and, for its peripheral/serum component, in a humanized xenograft model (elevated serum TARC and splenic Treg infiltration); direct confirmation of the serum readout in patients is still pending (Aoki et al. [6]). Corresponding systemic correlates for the CN913, STB, and CN2P subtypes are proposed as hypotheses requiring prospective paired tissue–blood validation (Section 8).

**Table 1:** Hodgkin and Reed–Sternberg (HRS) cells and major immune/stromal cell populations in classic Hodgkin lymphoma.

Cell Population	Principal Mediators/Markers	Biological Role in cHL	Clinical Significance
HRS cells	PD-L1, PD-L2, CD30, CD40, CCL17/TARC, CCL22, IL-6, IL-10, TGF-β	Orchestrate immune escape, chronic inflammation, and microenvironment remodeling [3,9,13]	Central therapeutic target; source of circulating biomarkers
CD4+ helper T cells	CD40L, ICOS, CXCR4/CCR4	Provide survival signals to HRS cells via cytokine and receptor interactions [18,19]	Contribute to immune-supportive niche
Regulatory T cells (Tregs)	FOXP3, CTLA-4, CCR4	Suppress antitumor immunity and maintain immune tolerance [20]	Prognostic significance depends on spatial localization
Exhausted CD8+/CD4+ T cells	PD-1, LAG-3, TIGIT, TIM-3	Impaired cytotoxic activity due to chronic antigen stimulation [19]	Reservoir for reinvigoration by PD-1 blockade
Tumor-associated macrophages (TAMs)	CD68, CD163, PD-L1, IL-10	Promote immune suppression, angiogenesis, matrix remodeling [21,22]	High density/adverse spatial niche linked to inferior outcome (conflicting results across studies) [21,22]
Dendritic cells	CD11c, CD83, HLA-DR	Antigen presentation and modulation of adaptive immunity [3]	Functional impairment may contribute to immune evasion
Natural killer (NK) cells	CD56, NKG2D	Innate cytotoxicity, suppressed by TGF-β [23]	Reduced number and function in cHL
Eosinophils/mast cells	IL-5 receptor, tryptase, histamine, VEGF	Tissue remodeling, angiogenesis, inflammatory amplification [3]	Particularly abundant in nodular sclerosis cHL
Cancer-associated fibroblasts (CAFs)	α-SMA, FAP, TGF-β, PDGF	Extracellular matrix remodeling, immune-cell trafficking barrier [3]	Potential therapeutic target; determinant of spatial organization

Major immune and stromal cell populations contributing to cHL pathogenesis. Rather than acting independently, these cellular components establish a dynamic, spatially organized ecosystem that promotes immune escape, chronic inflammation, and lymphoma progression. Statements are supported by the references indicated in the table; the clinical significance column summarizes observational findings unless stated otherwise.

**Table 2:** Emerging (not yet clinically validated) molecular subtypes of classic Hodgkin lymphoma defined by multidimensional tumor–immune architecture profiling (Aoki et al., 2026) [6].

Subtype (% of Cohort)	Genomic Features	TME/Spatial Features	Clinical Association
CST (31%)	CSF2RB mutations (cytoplasmic domain, truncating, E788* hotspot); TNFAIP3, MFHAS1, GNA13, BCL7A alterations; JAK2–STAT5 gain-of-function (functionally validated)	FOXP3+ Treg enrichment; significant TARC+HRS–CCR4+CD4+ T-cell interaction	Younger age; effects of CSF2RB mutations reversed by the JAK2 inhibitor pacritinib in cell-line and xenograft models (preclinical) [6]
CN913 (25%)	9p24.1 gain (JAK2, CD274, PDCD1LG2); 13q arm deletion; 6q23.3/1p36.32 deletion; KMT2D, TP53, KMT2A alterations	LAG-3+CD4+ T-cell enrichment; PD-L1+ macrophages near HRS cells; IFN-γ and NF-κB signaling	~79% EBV-positive (OR 6.89); reproduced in an independent ctDNA-based cohort (*p* < 0.001) [6]
STB (25%)	STAT6, B2M mutations (→ MHC-I loss); SOCS1, XPO1, IKBKB, EEF1A1 alterations	Immune-cold; fewer Granzyme B+ CD8+ T cells; TGF-β signature in HRS cells; galectin-9+HRS–TIM-3+ macrophage interaction	Trend toward inferior PFS; 40% of relapsed biopsies classified as STB [6]
CN2P (18%)	2p15 gain (REL, BCL11A); 12q13.3 gain; NFKBIA, IL4R, EGR1, FAS alterations; DNA-repair/TP53-target signature	PD-1+CD4+ T-cell enrichment near HRS cells; PD-L1+HRS–PD-1+CD4+ T-cell interaction (exhausted phenotype)	Older age, confirmed in validation cohort (*p* < 0.0001) [6]

Data from Aoki et al. [6]. Discovery cohort n = 114 (NMF clustering on 80 genomic features); validated in an independent ctDNA-based cohort (n = 293). Percentages refer to the discovery cohort. PD-L1/PD-1/LAG-3 immune-checkpoint associations support HLGen as a candidate predictive-biomarker framework; therapeutic hypotheses beyond the experimentally validated CSF2RB–JAK2 axis remain to be prospectively tested.

**Table 3:** Comparison of the ctDNA-based H1/H2 classification and the HLGen (CST, CN913, STB, CN2P) classification of classic Hodgkin lymphoma.

Feature	H1/H2 Classification (Alig et al., 2024) [42]	HLGen Classification (Aoki et al., 2026) [6]
Sample source	Plasma cell-free/circulating tumor DNA (liquid biopsy)	HRS-cell-enriched frozen diagnostic biopsies (laser-capture microdissection/FACS); ctDNA used for validation
Cohort size	366 patients profiled	Discovery cohort n = 114 (103 treated with ABVD or equivalent); independent ctDNA validation cohort n = 293 (the cohort used by Alig et al.)
Genomic inputs	Targeted ctDNA sequencing–derived genomic features	Whole-exome/targeted sequencing; NMF clustering on 80 recurrently altered genes/loci (mutations and copy-number alterations)
Number of classes	2 (H1, H2)	4 (CST, CN913, STB, CN2P) plus “UNCLASS” when classifier confidence is low
Additional data layers	Transcriptional and immunological profiles; DNASE1L3-dependent ctDNA shedding	GeoMx spatial transcriptomics of CD30+ HRS cells; imaging mass cytometry; functional modeling of CSF2RB mutations
Validation	Large single cohort; independent prospective validation not yet reported	Independent ctDNA cohort reproduced all four classes; no prospective validation
Clinical associations	Characteristic clinical and prognostic correlates reported	CN913–EBV positivity; CST–younger and CN2P–older age; no significant PFS difference (trend toward inferior PFS in STB)
Therapeutic hypotheses	Truncating IL4R mutations dependent on IL-13 signaling, targetable with anti-IL-4Rα antibodies (preclinical)	CSF2RB–JAK2–STAT5 axis in CST, reversed by pacritinib (preclinical); other hypotheses untested
Clinical readiness	Investigational; not used to guide treatment	Investigational (HLGen is a research classifier); not used to guide treatment

ABVD, doxorubicin/bleomycin/vinblastine/dacarbazine; ctDNA, circulating tumor DNA; FACS, fluorescence-activated cell sorting; HRS, Hodgkin and Reed–Sternberg; NMF, non-negative matrix factorization; PFS, progression-free survival. Cohort details are those reported in the original publications [6,42].

**Table 4:** Clinical correlates compatible with the proposed model of classic Hodgkin lymphoma as a systemic immunobiological disease.

Clinical Feature	Biological Basis	Clinical Relevance
B symptoms (fever, weight loss, night sweats)	Elevated cytokines (e.g., IL-6, TNF-α, TARC/CCL17) produced by HRS cells and immune infiltrate [50]	Associated with disease burden and adverse prognosis
Extranodal involvement (bone marrow, liver, lung)	Tumor-driven immune modulation and trafficking of immune cells to distant tissues [48,49,50]	Defines advanced-stage disease and influences risk stratification
Peripheral immune alterations	T-cell exhaustion, Treg expansion, myeloid skewing (MDSC-like phenotypes) [45,46,47]	Potential biomarkers for disease monitoring and therapeutic response
Circulating biomarkers (ctDNA, cytokines)	Release of tumor-derived DNA and soluble mediators into circulation [44]	Enables minimal residual disease detection and early relapse identification
Heterogeneous treatment response	Variability in tumor biology, molecular subtype, and immune competence [45,46]	Guides need for risk-adapted and personalized therapy
Patterns of relapse (including new sites)	Residual systemic disease and clonal evolution [44]	Supports integration of molecular monitoring strategies

Adapted and updated from Carbone & Gloghini [5]. These are clinical and observational associations and do not by themselves prove tumor-driven systemic immune reprogramming.

## Data Availability

No new data were created or analyzed in this study.

## Abbreviations

The following abbreviations are used in this manuscript:

AI	Artificial intelligence
ABVD	Adriamycin (doxorubicin), Bleomycin, Vinblastine, Dacarbazine
A-HIPI	Advanced-stage Hodgkin Lymphoma International Prognostic Index
ASCT	Autologous stem-cell transplantation
cHL	Classic Hodgkin lymphoma
ctDNA	Circulating tumor DNA
EBV	Epstein–Barr virus
GVD	Gemcitabine, vinorelbine, liposomal doxorubicin
HRS	Hodgkin and Reed–Sternberg
ICC	International Consensus Classification
IPS	International Prognostic Score
LMP1	Latent membrane protein 1
MDSC	Myeloid-derived suppressor cell
MRD	Minimal residual disease
MTAP	Methylthioadenosine phosphorylase
NF-κB	Nuclear factor kappa-light-chain-enhancer of activated B cells
NMF	Non-negative matrix factorization
PD-1	Programmed cell death protein 1
PD-L1	Programmed death-ligand 1
PFS	Progression-free survival
TAMs	Tumor-associated macrophages
TARC	Thymus and Activation-Regulated Chemokine
TMTV	Total metabolic tumor volume
TME	Tumor microenvironment
Tpex	Progenitor exhausted T cells
Tregs	Regulatory T cells
WHO	World Health Organization
